# Advances in Aeroengine Cooling Hole Measurement: A Comprehensive Review

**DOI:** 10.3390/s24072152

**Published:** 2024-03-27

**Authors:** Shuyan Yan, Junkai Shi, Guannan Li, Can Hao, Ying Wang, Hao Yu, Weihu Zhou

**Affiliations:** 1 Institute of Microelectronics of the Chinese Academy of Sciences, Beijing 100029, China; yanshuyan22@mails.ucas.ac.cn (S.Y.);; 2 University of Chinese Academy of Sciences, Beijing 100049, China; 3 China Aviation Planning and Design Institute (Group) Co., Ltd., Beijing 100120, China

**Keywords:** film cooling, cooling hole, optical measurement, online digital measurement, simultaneous measurement of multiple parameters

## Abstract

Film cooling technology is of great significance to enhance the performance of aero-engines and extend service life. With the increasing requirements for film cooling efficiency, researchers and engineers have carried out a lot of work on the precision and digital measurement of cooling holes. Based on the above, this paper outlines the importance and principles of film cooling technology and reviews the evolution of cooling holes. Also, this paper details the traditional measurement methods of the cooling hole used in current engineering scenarios with their limitations and categorizes digital measurement methods into five main types, including probing measurement technology, optical measurement technology, infrared imaging technology, computer tomography (CT) scanning technology, and composite measurement technology. The five types of methods and integrated automated measurement platforms are also analyzed. Finally, through a generalize and analysis of cooling hole measurement methods, this paper points out technical challenges and future trends, providing a reference and guidance for forward researches.

## 1. Introduction

With the continuous development of the aviation industry, the improvement of aero-engine performance has become a crucial issue with great attention in the modern aeronautical field. The turbine inlet temperature is one of the key technical indicators of aero-engines, and improve of turbine inlet temperature is an effective way to increase thrust and thrust-to-weight ratio. According to calculations, for each 55 °C increase in total turbine temperature, the engine thrust will increase by approximately 10% [1]. Nowadays, the turbine inlet temperature of the fourth-generation aero-engine, which has a thrust-to-weight ratio of around 10, has reached approximately 2000 K [2]. A proposed method from the Beihang University Comprehensive Thermal Management Team indicated that the turbine inlet temperature has even achieved 2400 k. This advancement can increase the theoretical speed range by 156%, reduce fuel consumption by 15%, and enable higher Mach numbers and longer flight range [3].

**Figure 1:**
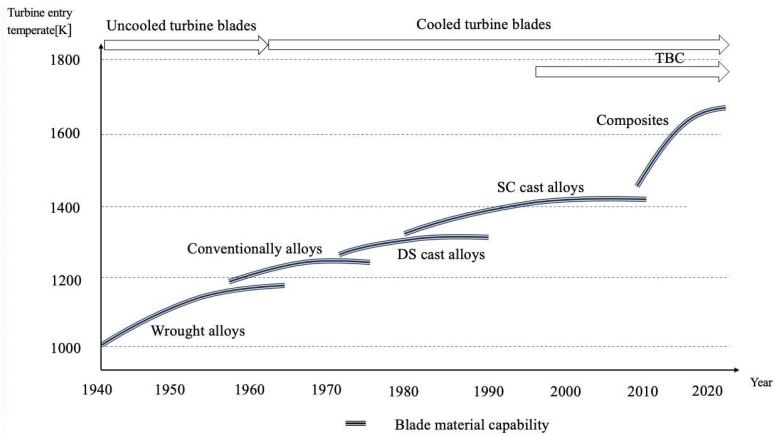
Development trend of aero-engine blade material.

The extremely high temperature means hot section components face harsh operating conditions, especially for turbine blades. To address this issue, the advanced alloy material with thermal barrier coating has been developed to process better heat resistance capacity [4]. However, fourth-generation single-crystal alloy material has a maximum temperature tolerance of about 1450 K [5], which cannot meet the required operational condition of approximately 2000 K. To overcome the heat resistance limitations of blade materials and avoid blade failure due to excessive operating temperature, thermal protection technology should be integrated in the blade’s design and manufacturing. This integration fills the temperature gap and ensures blades maintain excellent reliability and service life in harsh operating conditions. Figure 1 illustrates the development of blade materials in response to the increase turbine temperatures.

**Figure 2:**
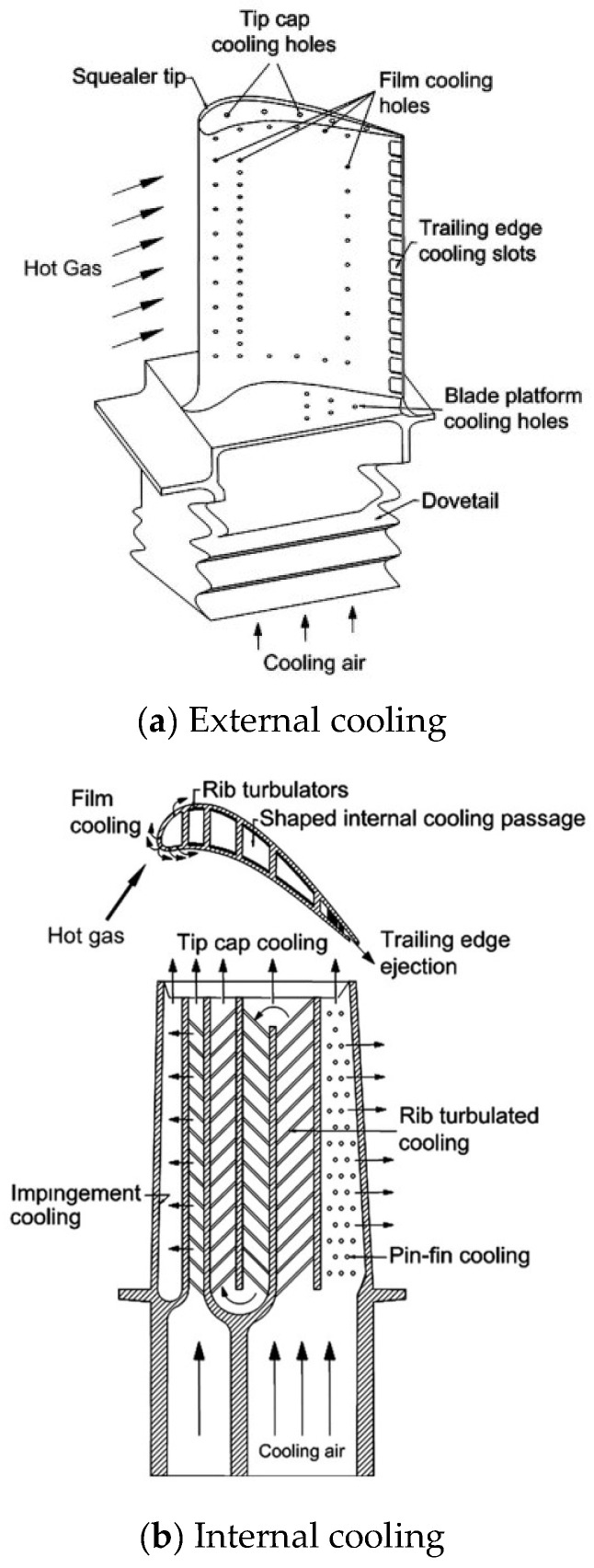
Schematic diagram of turbine blade cooling technologies [6].

**Figure 3:**
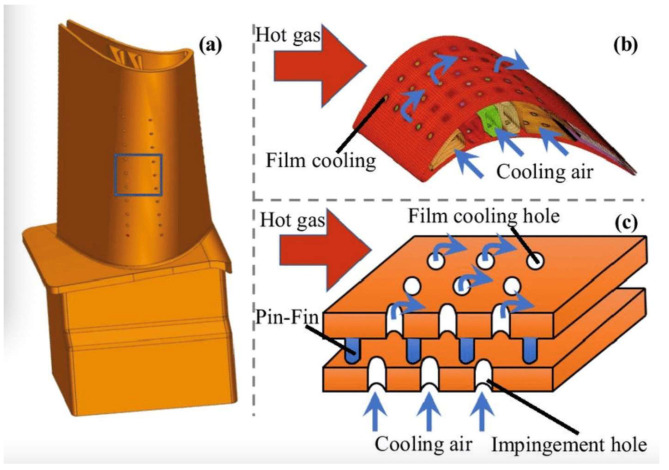
Schematic diagram of film cooling principle [7]. (**a**) Turbine blade model; (**b**) Schematic diagram of film cooling principle for blade; (**c**) Schematic diagram of film cooling principle for cooling hole.

In addition to high temperature-resistant materials and thermal barrier coating, thermal protection technologies mainly include air cooling technology which can be generally divided into two types: internal cooling and film cooling (external cooling). Figure 2 presents a schematic diagram of turbine blade film cooling technology [6], including external and internal cooling. In internal cooling, the cool airflow is guided through the internal passage to enhance the heat transfer. In film cooling, cool airflow expels from the internal passage and cooling holes and is applied to the blade surface as the protection layer, shielding the blade from the impact of high-temperature air. And Figure 3 presents a schematic diagram of principle of film cooling [7].

**Figure 4:**
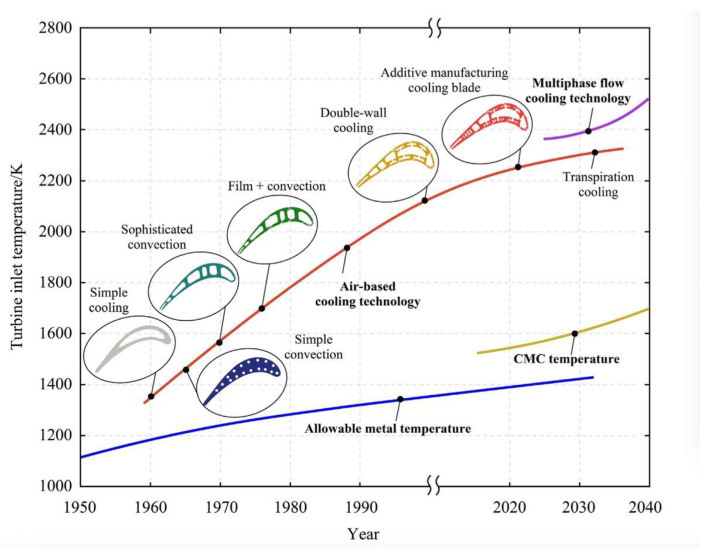
Development trend of cooling methods against the turbine inlet temperature [11].

Moreover, the essential of the film cooling technology is to have numerous cooling holes (with diameters about 0.3 to 0.5 mm and depth-to-diameter ratios up to 10:1) distributed as rows along the leading edge, pressure and suction side, and blade tip region to generate air circulation [8,9]. According to the literature reports, thermal barrier coatings can reduce the temperature of turbine blades’ surface by around 100 to 150 K. In comparison, film cooling technology can decrease the temperatures from around 400 to 500 K with more effective cooling performance [10]. Since 1970, film cooling technology has been designed into turbine blades and has become an effective method of thermal protection. Also, this method can be used in conjunction with other cooling methods. Figure 4 presents the development of blade cooling methods since 1960 [11].

The geometrical structures of cooling holes, including axial angle, diameter, orifice shape, spacing, and hole positional accuracy, are key indicators of film cooling technology which influence the effectiveness of cooling performance [12,13,14,15,16,17]. To meet measurement requirements for cooling hole geometrical parameters, it is vital to quantitatively assess all cooling hole characteristics by an efficient and precise method. Measurement results of critical indicators are helpful to understand the relationship between the geometrical structure of cooling holes and the effectiveness of film cooling technology. As well as this, researchers can further ensure safe, reliable operation and extend the service life of aero-engines according to the measurement results. 

In this paper, some of the literature related to the cooling hole measurement are reviewed. The overall structure of this paper is presented as follows. Section 2 briefly describes the development of shaped cooling holes with their geometrical features and corresponding enhanced cooling performance. Section 3 summaries and compares common manufacturing processes of the cooling hole. Section 4 focuses on the measurement technologies for the cooling holes. The key measurement indicators, traditional measurement methods, and principles of digital measurement technology are detailed, respectively. Further, the advantages, disadvantages and applied scenarios of each digital measurement technology are analyzed and compared. Finally, Section 5 gives the conclusion and outlook for future work.

## 2. Development of Shaped Cooling Hole Geometrical Design

The most common type of cooling hole is the cylindrical hole, which can be manufactured by mature and low-cost methods, such as laser processing and electrical discharge machining (EDM). With the development of film cooling technology, more and more novel shaped cooling holes have been utilized to enhance the efficiency of cooling performance when compared to the cylindrical hole [18,19,20,21,22,23].

**Figure 5:**
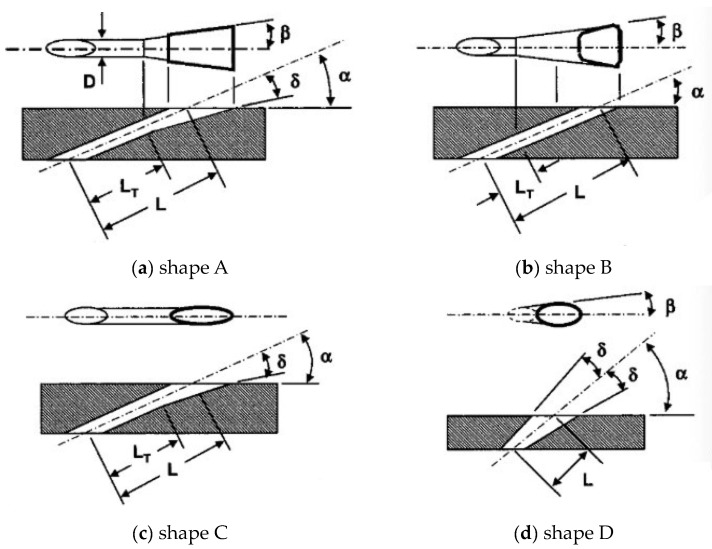
Geometries for 4 types of shaped cooling hole [19].

In 2005, Bunker [19] categorized the geometrical features of early shaped cooling holes into four types, as shown in Figure 5. The projection of the hole axis on the blade surface is considered as the longitudinal direction, and then its orthogonal direction is considered as the lateral direction. The cooling hole of shape A is the classic fan-shaped form, with both lateral and longitudinal expansion on the outlet area (angle β and angle δ). The cooling hole of shape B contains only lateral expansion on the outlet area (angle β), while the cooling hole of Shape C contains only longitudinal expansion on the outlet area (angle δ). Shape D is the conical shape hole, expanding equally in all directions from the inlet to the outlet, centered around the hole axis. 

Shaped cooling holes mainly adopt the design concept involving expanded outlets. The expansion of the outlet area facilitates the diffusion of the airflow to achieve a lower blowing ratio, reduce aerodynamic loss, and a larger cooling air coverage area, which is beneficial to enhancing cooling performance [20]. 

References [21,22,23] evaluate the cooling performances of various shaped cooling holes. Liu [23] concluded the improvements in the cooling performance of various shaped cooling holes against cylindrical cooling holes in Table 1. However, shaped cooling holes require more complex manufacturing processes, demanding higher precision and more advanced manufacturing technologies. Also, compared with the cylindrical hole, shaped hole involves more geometrical features can result in measurement difficulties. Finally, as the outlet area of cooling holes expands, the spacing between each hole gets larger. This means that there are fewer shaped holes for the definite blade surface area than normal cylindrical holes. The reformation of shape poses a challenge for the arrangement of cooling holes.

## 3. Development of Cooling Hole Manufacturing Processes

### 3.1. Cooling Hole Manufacturing Processes

The main manufacturing processes for cooling holes include electrical discharge machining (EDM), electrohydrodynamic (EHD) jet drilling, and laser drilling. 

Currently, EDM is the most widely used process with mature technology. The principle of EDM is removing material through electro-erosion which is caused by spark discharge between the positive and negative electrodes of the tool and the workpiece, whereas due to the limitation of the material properties, the ceramic material has poor electrical conductivity. It is particularly challenging to process ceramic thermal barrier coatings. Also, EDM is characterized by low efficiency and low precision. The noise, smoke, and harmful gases generated in manufacturing are potentially hazardous to operators [24].

The principle of EHD jet drilling method is based on the micro holes manufacturing using a metal tube electrode. The workpiece is connected to the positive electrode, while a metal tube in a glass nozzle is connected to the negative electrode, and then electrolyte solution is ejected from the glass tube through a high-voltage electric field. Eventually, excess material from the positive electrode metal workpiece is removed under the influence of an electric field [25]. This method is not a thermal manufacturing method. Therefore, it can lead to better surface quality and lower roughness without microcracks or recast layers on the inner walls of the cooling holes. However, a drawback of this method is the difficulty in controlling the shape of cooling holes during the electrolytic etching process. Compared to the other two methods, its application is less widespread.

The principle of the laser drilling method is removing excess material by heating and melting with a high-energy light beam [26]. This method is known for its high precision, high efficiency, and wide applicability of materials, making it a key area of current research for the cooling hole manufacturing process.

Considering the trade-off against economic benefits and production quality, nanosecond laser drilling and electrical discharge machining (EDM) are common choices in engineering production. However, both methods are thermal processes, which create heat-affected zones on the inner walls of cooling holes, leading to microcracks and recast layers. These effects can significantly impair the cooling efficiency, overall reliability and service life of the turbine [27]. To mitigate thermal damage, important progress has been made in cooling technologies, specifically for cooling hole manufacturing. General Electric (GE) company in the USA, in collaboration with SYNOVA corporation in Switzerland, developed the water-assisted laser method. Water helps to lower the temperature in the manufacturing area, wash away debris, and guide the laser beam to the manufacturing target [28]. Similar research on water-assisted laser methods has been conducted in China, with Zhang Wenwu of the Chinese Academy of Sciences proposing a method [29]. These technologies effectively reduce thermal damage and enable high-quality manufacturing of cooling holes.

**Figure 6:**
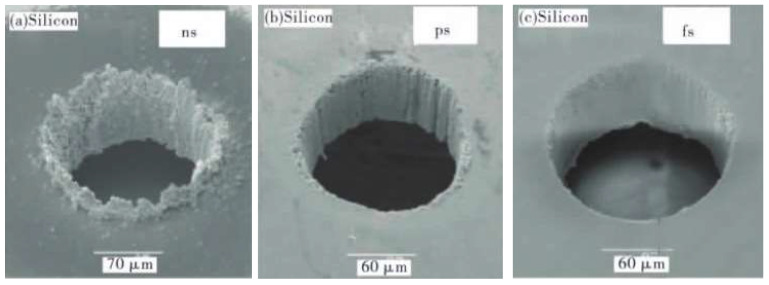
Manufacturing quality against three types laser drilling method [33]. (**a**) Micro hole drilled by nanosecond laser. (**b**) Micro hole drilled by picosecond laser. (**c**) Micro hole drilled by femtosecond laser.

On the other hand, drilling processes continuously evolve, with researchers developing methods such as femtosecond laser drilling [28,30] and compound laser drilling with varying pulse width [31]. To some extent, these methods have improved issues related to microcracks and recast layers, reduced the harm caused by the heat-affected zone, and enhanced precision and efficiency in processing. However, these drilling processes cannot completely eliminate the effects of thermal manufacturing, indicating that manufacturing processes of cooling holes should be optimized further [32]. Figure 6 displays three types of micro holes drilled by nanosecond, picosecond, and femtosecond lasers [33].

### 3.2. Difficulties in Cooling Hole Manufacturing

Currently, there are several technical challenges that exist in the manufacturing of cooling holes.

**Figure 7:**
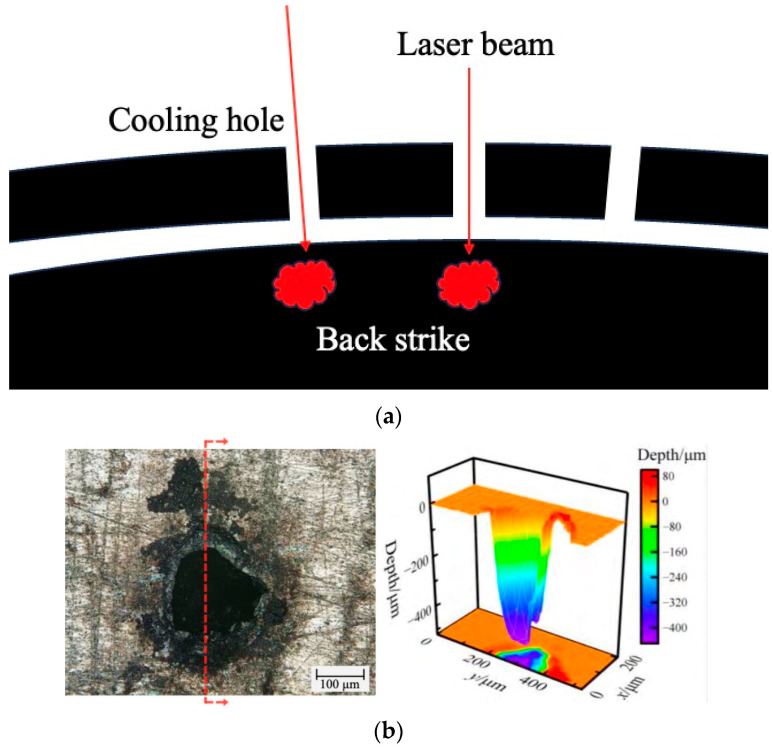
Schematic diagram of back strike. (**a**) Principle of back strike. (**b**) Example of back strike [35].

Back strike is a common issue during the laser drilling process. Due to immature manufacturing parameters, hollow and complex structure of blades, and other factors, the control system is unable to accurately identify the location and timing of drilling. These factors may result in incomplete perforation, creating blind cooling holes, or over-penetration even after the hole is made, leading to melting or scorching on the inner wall of cooling hole and damage to the internal cooling paths of blade [34], as shown in Figure 7 [35]. Due to the complexity of internal cooling paths in the blade, conventional methods cannot observe the internal condition, making it difficult to detect back strike. Hence, it is necessary to implement quality control during the manufacturing of the cooling holes to improve manufacturing precision.

Drilling holes in blades covered with a thermal barrier coating presents another challenge in manufacturing cooling holes. For the blades with a thermal barrier coating, the conventional sequence of the hole manufacturing process is “drilling before coating”. This method can result in cooling hole shrinkage, where the coating may obstruct or cover the orifices, causing a reduction in orifices area, diminishing the airflow capability, and even leading to the clogging of the cooling holes. According to the literature reports, the shrinkage rate of this method can be 15% to 20%, with the hole diameter reducing from 0.35–0.4 mm (before coating) to 0.25–0.3 mm (after coating). Additionally, the drilling process may damage the material structure around the edges of the orifice, resulting in fatigue cracks, which ultimately affect the bonding strength between the thermal barrier coating and the blade surface, as well as their impact resistance capabilities [36].

“Drilling after coating” is a new developing trend. The SYNOVA corporation [37] proposed a method that combines abrasive water jet machining and EDM, applying the “drilling after coating” technology to alloy blades. But differences in thermal expansion coefficients, toughness, stiffness, interface geometric structures and other material properties between the thermal barrier coatings and the alloy materials can result in potential issues such as interlayer tearing and cracking [38], impacting the safe service of the blades.

In response to these challenges in the cooling hole manufacturing processes, engineered solutions are unavailable nowadays. It is necessary to improve manufacturing precision to avoid or mitigate the risks associated with the above issues.

## 4. Development of Cooling Hole Measurement Technology

### 4.1. Key Quality Indicators for Cooling Hole

The key quality indicators for cooling holes primarily include:

1. Hole Diameter: The diameter of hole orifice, with a general tolerance of 0.10 mm.
2. Hole Positional Accuracy: the hole geometric position and hole spacing. The geometric position is the coordinate value of the point where the hole’s axis intersects the blade profile in the blade coordinate system. The tolerance for position accuracy is generally between 0.10–0.15 mm.
3. Hole Axis Orientation: The angular tolerance of the hole axis is generally within ±1° [39]. Currently, there are no explicit design standards or technical requirements for the axis angle [40].
4. Orifice Shape: orifice shapes have two main types: circular orifices and shaped orifices.
5. Internal Surface Roughness: The surface quality of inner wall is influenced by the manufacturing process. Presently, there are no specific numerical requirements.
6. Maximum Thickness of recast layer: thermal manufacturing processes induce thermal effects, forming a recast layer on the hole’s inner walls. The thickness of recast layer needs to be controlled. Presently, there are no specific numerical requirements.
7. Blind Hole Rate: Design specifications require that the blind hole (blocked hole) rate should be 0%, ensuring that each hole is fully open and functional for proper airflow distribution.

**Figure 8:**
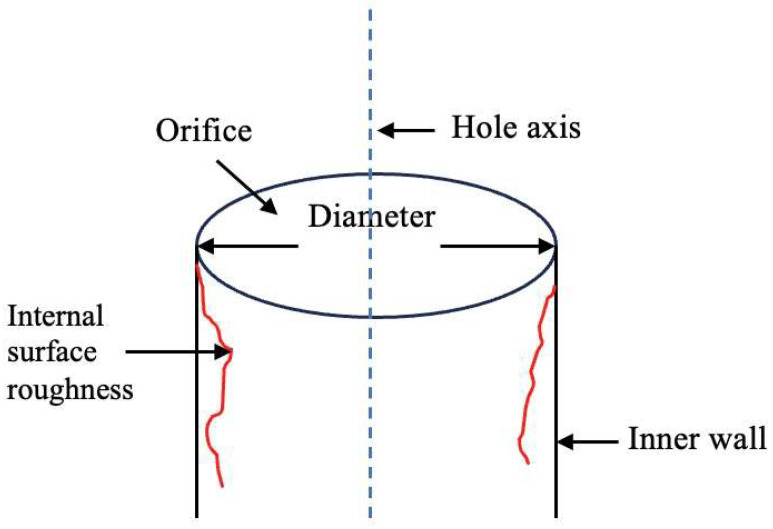
Schematic diagram of discrete cooling hole quality indicators.

The schematic diagram of discrete cooling hole quality indicators is demonstrated in Figure 8.

### 4.2. Cooling Hole Measurement Technology

#### 4.2.1. Traditional Measurement Method

Currently, the measurement of cooling holes often relies on manual inspection by quality checkers, including plug gauges, visual comparison, and water flow tests.

1. Plug gauge method

**Figure 9:**
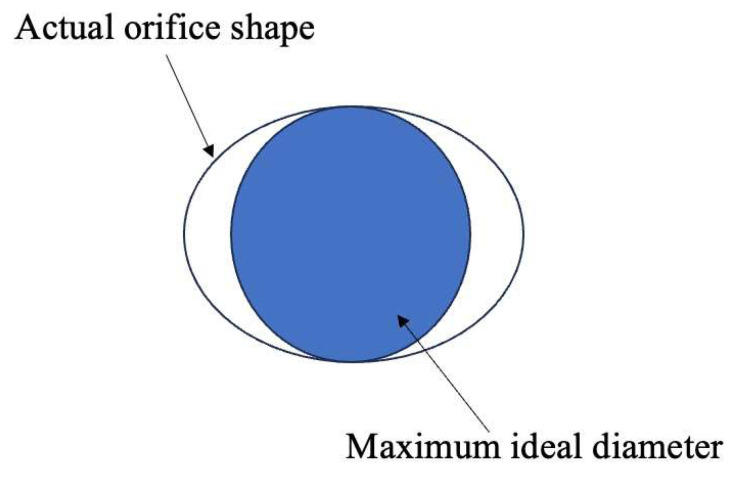
Schematic diagram of plug gauge measurement.

Quality checkers use plug gauges with different diameters, inserting them into a cooling hole to approximate the internal diameter and thus measure the diameter of the hole. This method is extremely slow and risks the plug gauge breaking inside the hole if mishandled, potentially resulting in scrap. Due to the manufacturing process of cooling holes, the actual internal surface has high roughness, poor roundness, and taper shape. The plug gauge can only measure the maximum ideal diameter in such cases, the schematic diagram is shown in Figure 9.

2. Sample Visual Comparison Method

This method relies on the visual judgment of quality checkers. They compare the blade with the standard sample to judge the positional accuracy, orifice shape, and diameter of the cooling hole. Presently, there is no unified national calibration standard for calibrating the standard sample blades used in visual comparisons, nor are there instruments to calibrate the positional accuracy of cooling holes in standard samples. It is challenging to use standard blade samples for quantitative traceability or data transfer [40].

3. Water Flow Method

This method involves injecting water inside the blade and visually observing whether the cooling holes allow water to pass through and whether the flow value is similar. This method depends on the blade’s internal cooling path design and does not apply to all blades, making it less common than the other methods.

The measurement methods commonly used in engineering scenarios rely heavily on manual operation and invasive inspection techniques. Subjective manual judgments can only provide qualitative indicators. They cannot quantitatively evaluate critical quality indicators simultaneously, such as positional accuracy, hole axis orientation, hole diameter, and blind hole verification. As for the manufacturing quality of the internal surface of cooling holes, including issues like microcracks, recast layers, and thermal barrier coating defects, no corresponding measurement methods are currently available.

The automation and intelligence levels of traditional measurement methods urgently need improvement. By utilizing automated data collection and processing, it is possible to reduce manual intervention, thereby increasing efficiency and the accuracy and precision of measurement results.

#### 4.2.2. Digital Measurement Method

1. Probing Measurement Technology

**Figure 10:**
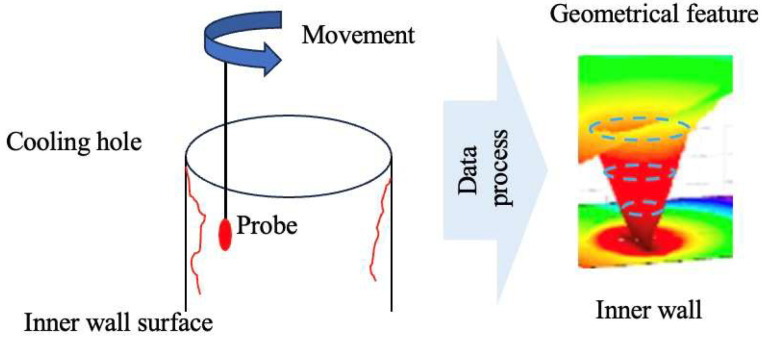
Working mechanism of probing measurement method.

**Figure 11:**
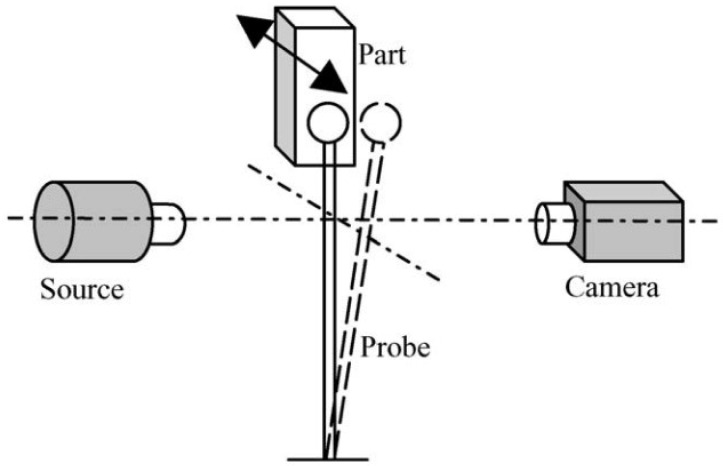
Measurement principle [45].

Researchers have taken advantage of miniature-sized probes for in-depth micro-hole measurements, including fiber probe technology [41,42,43,44,45] and capacitive probe technology [46,47,48,49,50,51]. The working mechanism of this method is presented in Figure 10. Salah crafted a rotational wire probe using stainless steel wire and a microtube, employing an acoustic emission device to perform contact detection by approaching and impacting the inner walls of cooling hole [41]. This method is utilized for measuring the diameter and roundness of the holes. Their experiment successfully measured the micro holes with diameters less than 1 mm and depth-to-diameter ratios of approximately 10:1, obtaining 3D profiles of the inner walls. Cui Jiwen [42] developed a twin Fiber Bragg Grating (FBG) probe for measuring large depth-to-diameter ratio micro holes. This design achieved multidimensional tactile perception along the *X*-axis and *Z*-axis while guiding the optical signal through the probe [43]. The design mitigates shadow effects to some extent, and the probe, with a diameter of less than 100 μm, is suitable for measuring micro holes in various industries. Building on this research, Feng Kunpeng [44] integrated the FBG probe with a measuring machine and introduced a data processing method with transformation of the signal domain and multiple fitting, enhancing the measurement accuracy of micro-hole diameters. Muralikrishnan [45] employed fiber deflection probing technology (FDP), integrating the fiber probe with a coordinate measuring machine to measure the diameter and shape of micro holes, achieving a measurement uncertainty of 0.07 μm. This technology can measure micro holes with depth-to-diameter ratios up to 20:1. However, the accuracy depends on the alignment of the probe with the hole axis and the machine axis, as there is no established reference standard for measurement. Figure 11 displays the measurement principle.

Ma Yuzhen [46,47] researched the capacitive probe measurement method for micro holes. To measure the diameter of deep and angled holes, they developed a non-contact capacitive probe that measures the gap between the probe electrode and the hole’s inner wall. They proposed a hole axis fitting algorithm that combines the projection and least squares fitting. Experiment results confirm that the measurement data maintains consistent accuracy for holes with a depth-to-diameter ratio exceeding 10:1 and is not affected by where the probe enters the hole. Sun Xuan [48] established a micro hole diameter measurement system based on a coaxial cylindrical capacitive sensor. By identifying the central axis of the sensor and the micro hole using a charge-coupled device (CCD) camera and aligning the probe with the hole center using a movement system, then driving the capacitive probe into the hole and measuring micro holes with a depth-to-diameter ratio of 13:1. This system can measure the internal diameter at any depth within the hole, with a standard deviation 0.167 μm. Lee Neville [49] introduced a low-cost capacitive probe hole measuring system, determining the center position of the signal by locating the position of the minimum capacitive signal. Bian [50] developed a specialized hole diameter measurement system based on spherical scattering electric field technology. This system converts the tiny gap between a detection sphere on the probe and the test piece into an electrical signal, enabling non-contact, nanoscale resolution measurements of hole diameters.

Li Qi [8] utilized the principle of laser interferometry to design a cooling hole measurement system based on a laser rangefinder sensor. Guided by a digital blade model, the coordinate measuring machine (CMM) drives the probe into the cooling hole at various depths. The sensor acquires measurement data, which is processed by specialized software to determine the diameter of cooling holes and any position deviations.

In addressing the micro-probe measurement methods, researchers predominantly integrate CMMs with probes, controlling the probe to follow a planned path and scan the inner wall of cooling holes. These approaches offer high reliability and are not influenced by the hole depth or the inner wall’s characteristics, allowing precise measurements for critical areas. However, these methods cannot provide information on the positional accuracy or minor damages. These notable limitations include low efficiency, not being applicable for measuring curved holes, the potential for causing damage to the sample surface, and the inability to meet the demands of large-scale industrial measurements.

2. Optical Measurement Technology

**Figure 12:**
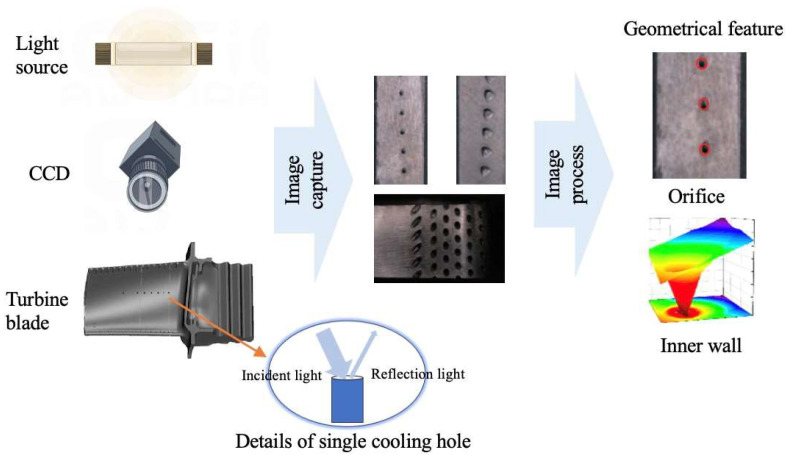
Working mechanism of optical measurement method.

Optical measurement technology represents a principal approach for non-contact measurement of cooling holes, encompassing machine vision, 3D reconstruction, stripe pattern projection, and luminous flux methods. The non-contact character of optical methods enables in situ measurement capabilities. Additionally, by not physically interacting with the object, these technologies offer significant advantages in preventing any potential damage to cooling holes during the measurement process. The working mechanism of this method is presented in Figure 12.

**Figure 13:**
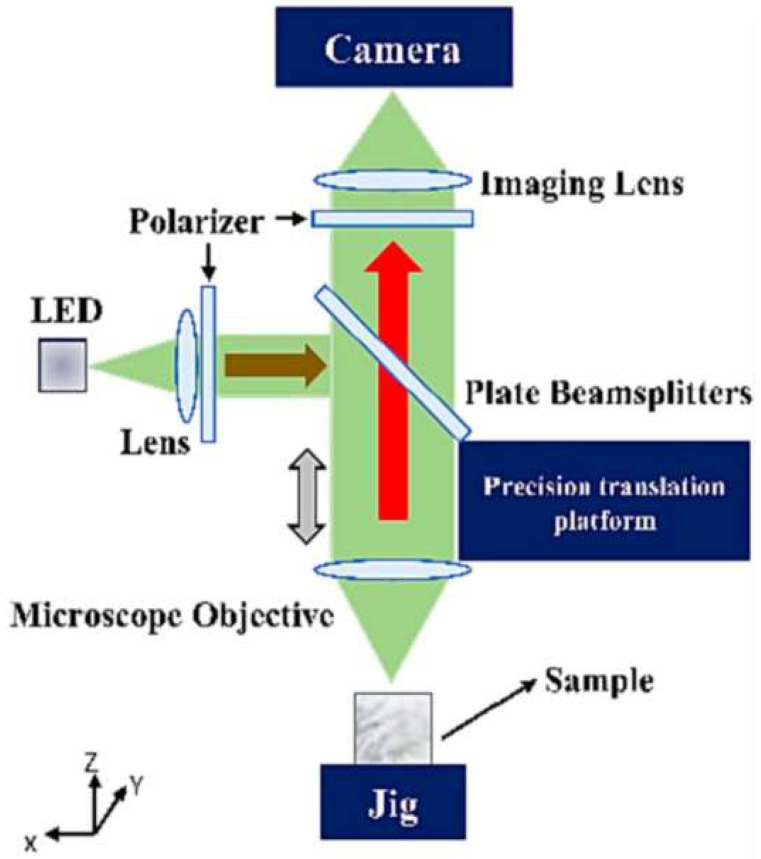
Principle of optical measuring system [52].

**Figure 14:**
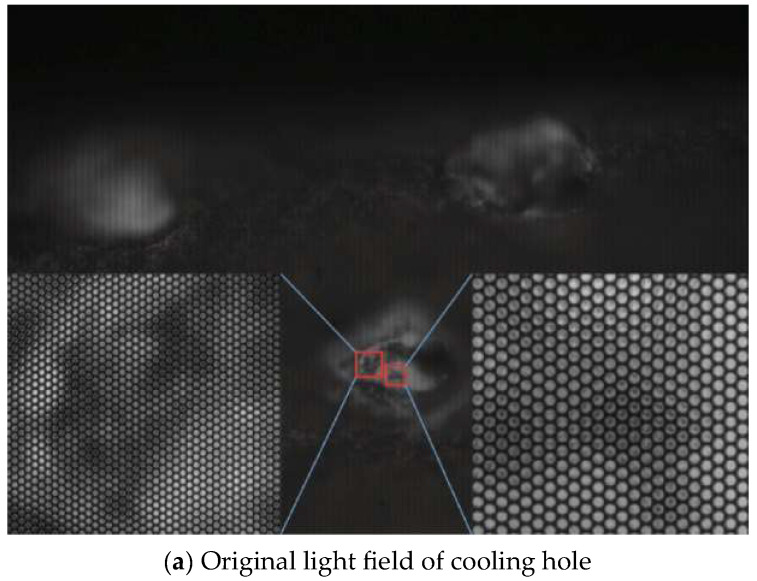

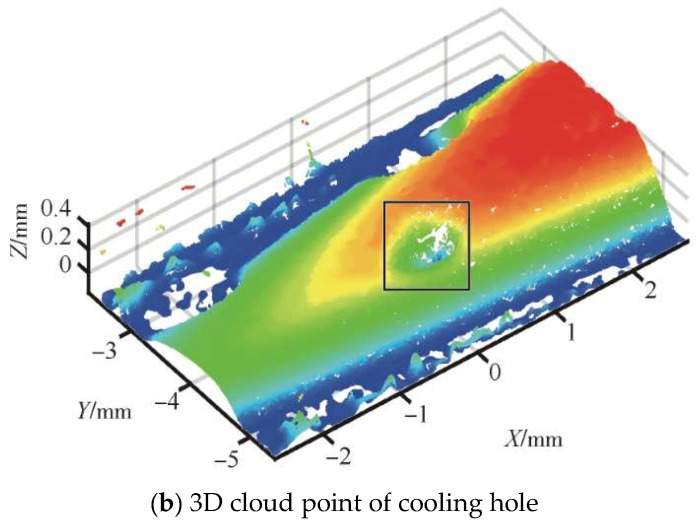
Experimental results of cooling hole [53].

Cheng Yuqi from Huazhong University of Science and Technology utilized a stereo vision 3D reconstruction technology to measure the diameter of cooling holes. The experiment results indicate that the evaluation error in measuring diameters is within 0.05 mm. This method allows simultaneous measurement of multiple cooling holes, significantly enhancing measurement efficiency [51]. Li Lei from Xi’an Jiaotong University [52] developed a cooling hole measurement method based on microscopic image sequence topographical reconstruction. A new measuring operator was established to measure the focus of the cooling hole image sequence. The reconstructed models obtained by this method show a standard deviation ranging from 0.007 mm to 0.018 mm. For cooling holes with a depth-to-diameter ratio close to 5:1, the absolute error in diameter is less than 0.01 mm. Figure 13 demonstrated measurement system. Zhao Yuanyuan from Shanghai Jiaotong University [53] employed a light field camera to capture sub-aperture images of cooling holes. Epipolar plane images (EPI) was generated from sub-aperture images and convert depth information into 3D point cloud data through EPIs. This method captures the 3D point cloud of cooling holes in a single exposure, greatly enhancing the measurement efficiency of cooling holes and demonstrating the potential of light field cameras in the micro hole measurement area. Figure 14 presents the original light field image and 3D cloud point of cooling hole.

Munkelt [54] addressed the issue of thermal barrier coating potentially covering or filling cooling holes. An optical 3D scanning method based on the fringe projection principle was utilized to scan the blades, enabling the automatic detection of covered cooling holes and precise guidance for the laser drilling process. Xu Dongjing at Nanjing University of Aeronautics and Astronautics [55,56] proposed a method for measuring the geometric parameters of micro holes based on luminous flux. They established a mathematical model correlating the area of an orifice with the emitted luminous flux. A measured hole is non-compliant if the measured luminous flux does not align with the luminous flux values for standard holes. This method can meet the industrial measurement requirements, which is a variation rate of 5%. Still, it only provides qualitative assessments, lacking the capability for quantitative measurement of hole geometry parameters. Jin, from South Korea [57], studied through silicon via (TSV) used in semiconductor device packaging. TSVs have an aspect ratio of 20:1 and diameters ranging from 50 to 200 μm [58]. Jin used an optical comb of femtosecond pulse laser in the infrared range as a light source and, based on spectral resolved interferometry, achieved measurement for micro holes with a depth-to-diameter ratio of 7:1. However, this method cannot provide information on the 3D profile of inner walls [57]. Wu Chunxia [59] developed a near-infrared microscopic interferometry technology with aberration compensation for TSV inspection. This method enabled micro hole measurement with a depth-to-diameter ratio up to 6:1, including the depth and bottom surface morphology.

The above measurement methods primarily focus on finished cooling holes. However, in situ measurements during the manufacturing process of cooling holes can timely detect errors, allowing for adjustment and optimization of manufacturing parameters, enhancing manufacturing quality to its maximum and avoiding defective workpieces. Weifang Sun [7] implemented an in-situ measurement method during the drilling process by integrating an image-capturing device into a laser drilling machine with an image edge feature extraction algorithm, enabling the measurement of cooling holes’ diameter and roundness. The experimental results indicate that the absolute errors of the diameter and roundness are 0.05 μm and 11.13 μm, respectively. This method demonstrates the potential and feasibility of in situ measurements in the domain of cooling holes. Shetty [60] utilized a vision system to acquire the diameter and orifice shape of cooling holes, coupled with a collimating tube to illuminate the inner wall to determine the presence of hole bottom. This method enables real-time in situ measurement of the drilling depth and drilling speed.

3. Infrared Imaging Technology

**Figure 15:**
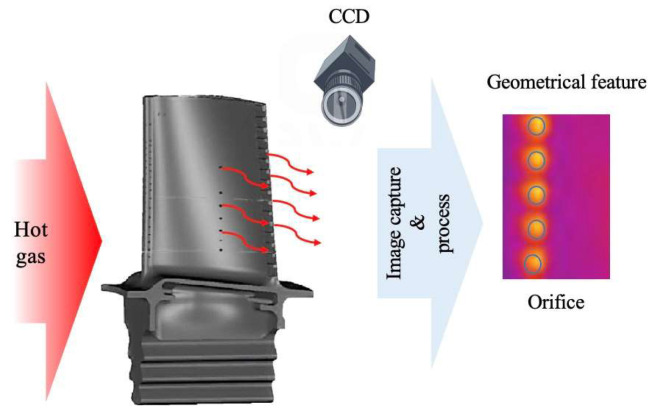
Working mechanism of infrared imaging measurement method.

**Figure 16:**
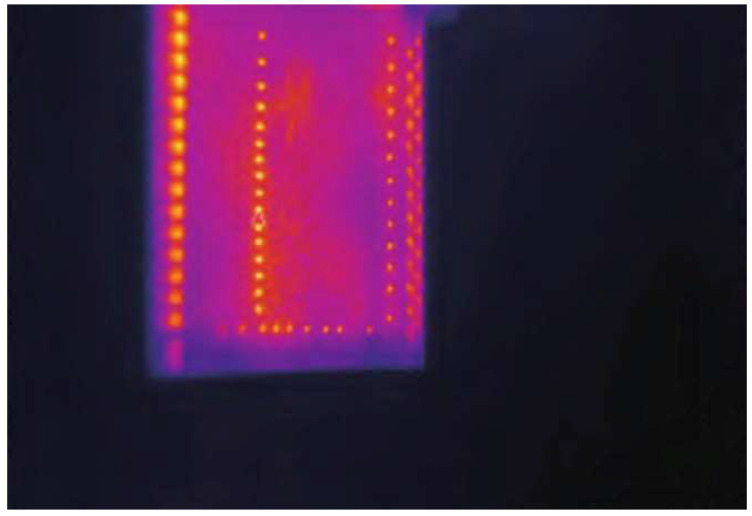
Infrared image of cooling hole [36].

Besides optical measurement technologies, researchers have exploited the principles of infrared imaging, generating heat within the blade and identifying the geometric characteristics of cooling holes through infrared images. The working mechanism of this method involves detecting and measuring the infrared radiation emitted by object. By using a detector to measure the difference in infrared radiation between the object and background, infrared images can be obtained [61]. The detailed working mechanism of this method is presented in Figure 15. Rosemau [62] developed a measurement system for cooling holes based on infrared imaging. This system cyclically heats and cools the blade, capturing infrared images of the thermal airflow effusing from the cooling holes. The temperature intensity change rate during the heating and cooling processes is utilized to assess hole quality. Experiment results indicate that the system achieved a 98.3% recognition rate for defective holes and a 99.7% recognition rate for qualified holes. This method allows for preliminary filtering of cooling hole quality through qualitative assessment, although it is limited in measuring precise geometric parameters due to its measuring principle. He Qing [63] proposed a high-pressure turbine cooling hole testing method based on infrared imaging principles, capable of identifying whether the holes are clear or blocked. Xia Kailong [36] optimized He Qing’s work by constructing an infrared measurement system, including a thermal imager, heat excitation source, turntable, and movement system. The system applies thermal excitation to cooling holes, captures infrared image sequences, and uses the canny algorithm and Hough circle transform function for image processing to calculate the diameters of cooling holes. Experiment results demonstrate that average deviations between the horizontal and vertical row of cooling holes and plug gauge are 4.40% and 2.32%, respectively. The infrared map of cooling hole is shown in Figure 16.

4. CT Scanning Technology

**Figure 17:**
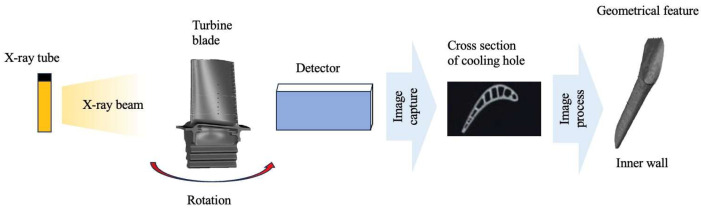
Working mechanism of computer tomography measurement method.

Industrial CT technology is a widely used non-destructive testing method that involves a series of X-ray measurements taken from different angles to generate cross-sectional images and 3D profiles of the scanned object, allowing users to see inside of the object without cutting. This technology applies to various aerospace components [64]. The working mechanism of this method is presented in Figure 17. Wang Wenhu [65] conducted measurements on blades using industrial CT scanner. They extracted a 3D point cloud of shaped cooling holes as key features. Through computational processing, they obtained critical parameters such as the size, shape, and contour of cooling holes.

**Figure 18:**
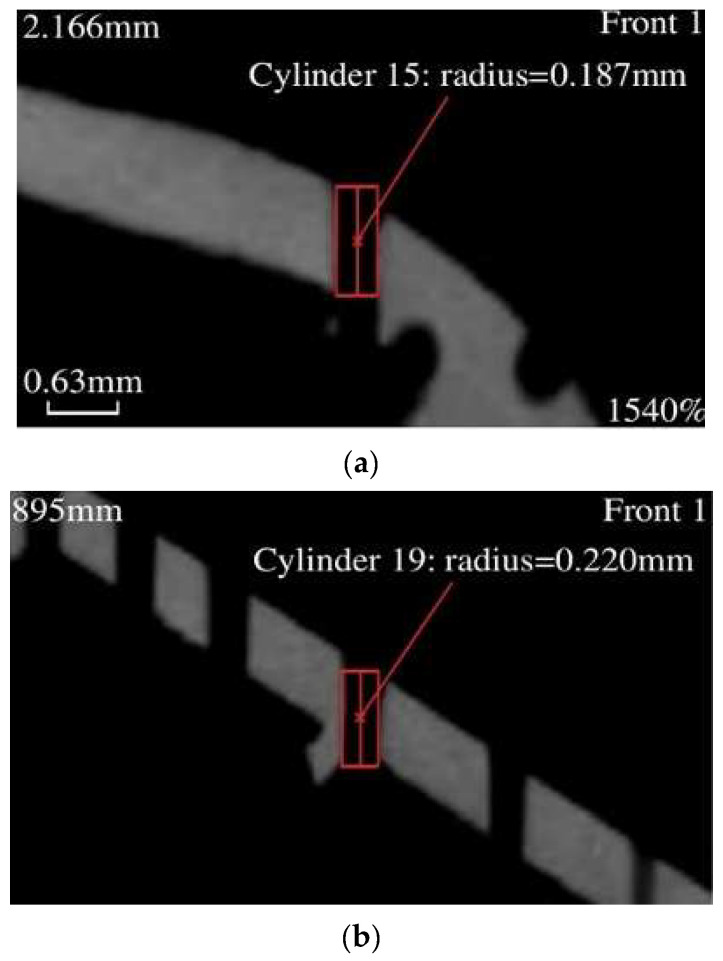
Blade profile image from industrial CT [66]. (**a**) Measurement results of squealer tip from industrial computer tomography (CT). (**b**) Measurement results of 2nd row cooling hole from industrial computer tomography (CT).

Jiang Qilin [66] conducted quality inspections on blades, using industrial CT to measure the cooling hole profiles. By examining cross-sectional scans, any internal wall intersections can be observed clearly. Figure 18 demonstrates a blade cross-section by industrial CT scan. Yang Zenan [67] utilized cone-beam CT technology to compare cooling holes manufactured with different manufacturing parameters and analyze their geometrical features and manufacturing quality. This work demonstrates the feasibility of using cone-beam CT technology to evaluate the quality of cooling holes.

5. Composite Measurement Technology

Due to the limitations of single-method measurements in obtaining comprehensive quality indicators of cooling holes, multi-sensor composite measurement methods have garnered attention.

**Figure 19:**
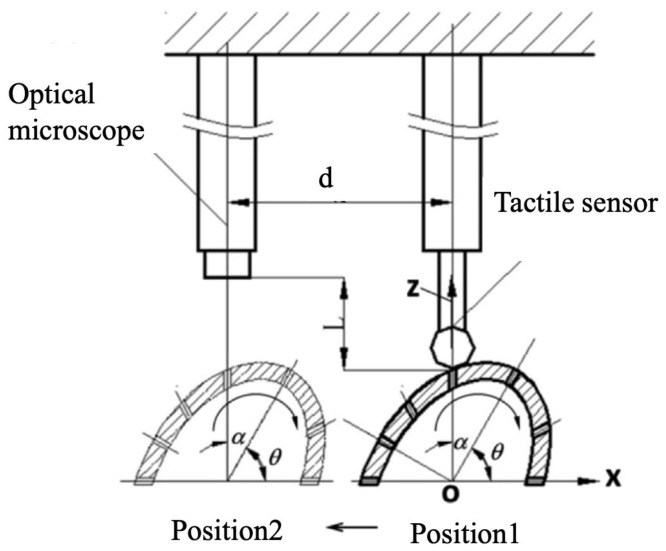
Dual-sensor autofocusing configurations [69].

Chen Xiaomei [68] researched measuring micro hole positions on complex curved surfaces. Traditional methods typically employ a single optical vision sensor, but the optical focusing function faces limitations due to the complexity of surfaces. Consequently, they proposed a dual-sensor autofocus method combining vision with tactile sensing, and experiment results indicate that for measuring micro holes with a diameter of 0.5 mm distributed on an elliptical cylinder, the focusing deviation ranges from −23 μm to +95 μm. This dual-sensor autofocus method proves to be a more accurate and reliable method for measuring micro holes on complex surfaces, detailed diagram is shown in Figure 19. Sui Xin from Changchun University of Science and Technology [69,70] developed a multi-sensor technology combining contact and non-contact measurements. They utilized a fiber probe to touch the inner wall along the hole, with a CCD recording the relative position of the inner wall and the probe. This method achieves the measurement of five parameters for micro holes, including cylindricity, diameter, roundness, taper, and straightness. Additionally, measurement results for cylindricity and taper demonstrate repeatability of 1.36 μm and 1.51 μm, respectively.

Stimpson [71] conducted research on the cooling performance of cooling holes produced via additive manufacturing. To assess these cooling holes, they employed a combination of Industrial CT and Scanning Electron Microscope (SEM), which allowed them to measure the geometric features of cooling holes and the roughness of inner walls. The integration of these two technologies provided a comprehensive analysis of both the macro-structural and micro-structural characteristics of cooling holes, offering insights into how additive manufacturing technology affects these critical components.

**Figure 20:**
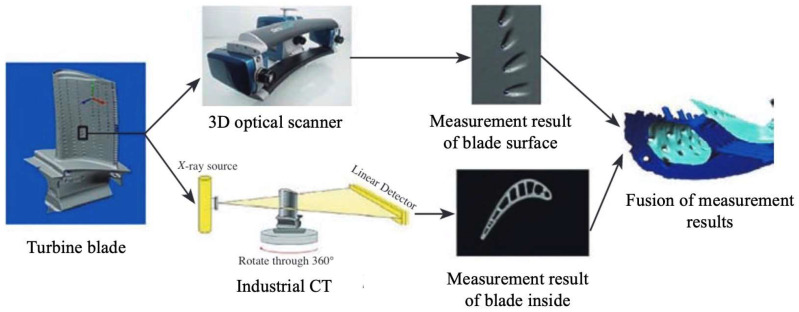
Technology roadmap of composite measurement system from SURVICE Metrology corporation [32].

NOVACAM company from Canada [72] developed the cooling hole inspection system, EDGEINSPECTTM, based on low coherence interferometry measurement technology. This non-contact inspection system is capable of acquiring high-precision 3D point cloud at speeds from 2100 to 100,000 points per second. The measurement results provided by this system include parameters such as the orifice shape, inner diameter, and the axial orientation of cooling holes. SURVICE Metrology, a company based in the United States, have integrated blade surface images from optical scanners with internal 3D profile from industrial CT, and analyzed the manufacturing accuracy of cooling holes [73]. The technology roadmap is illustrated in Figure 20. Additionally, General Electric (GE)company proposed a composite method combining CMMs with optical scanners to inspect the distribution of cooling holes in blades [74].

6. Automated Measurement Platform

**Figure 21:**
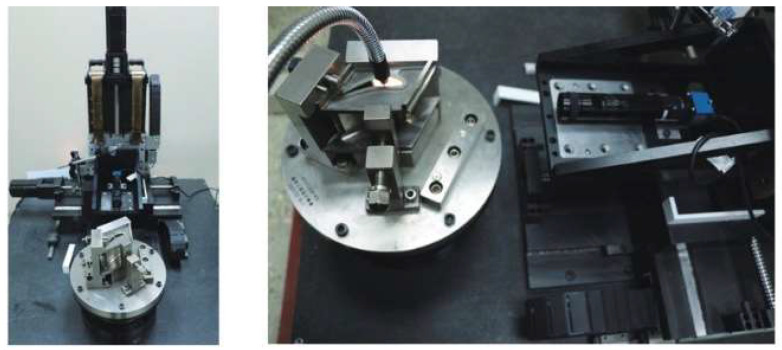
Diagram of detection device and imaging system [75].

To meet the measurement requirements of cooling holes, researchers have constructed an automated measurement platform that integrates machine vision and image processing methods to address practical inspection challenges and to study problems encountered in engineering applications. Bao Chenxing [75] designed and developed a 4-axis cooling hole measurement system based on CCD. This system uses a turntable to rotate the blade along a specific axis, and the CCD captures images of cooling holes during rotation without aligning a CCD with a hole. Further, the standard for cooling hole alignment is where the image of the cooling hole is a perfect circle. The hole axis and diameter are identified and calculated with Halcon17.12, an open-source image processing software. This system demonstrates the repeatability error of 0.2° for the hole axis and 0.1 mm for the hole diameter. Figure 21 illustrates the detection device and imaging system used in the experiment.

**Figure 22:**
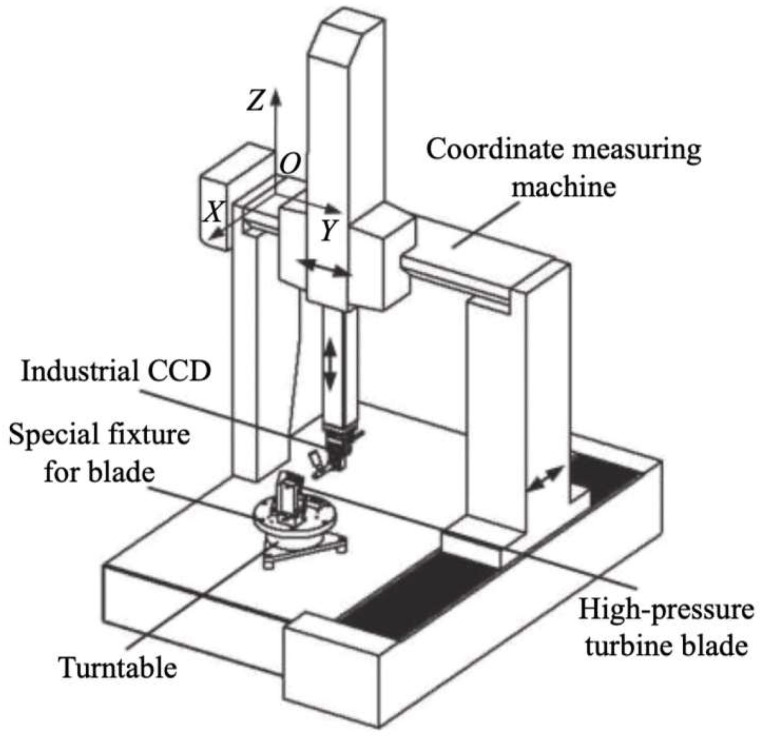
Schematic diagram of measurement a 4-axis vision coordinate measuring machine (CMM) [80].

Bi Chao and team from the Precision engineering Institute for aircraft industry conducted a series of studies [76,77,78,79,80,81,82] focused on cooling hole measurement and construction and design of the measurement system. These studies include establishing coordinate systems within the measurement systems and transforming measurement data from 2D coordinates in the image coordinate systems to 3D coordinates in global coordinate systems by mathematical approach [76]. Additionally, Bi constructed 4-axis and 5-axis visual measurement platforms using CMM, high-precision turntables, and CCD [39,77,78,79,80]. The 4-axis visual measurement platform captures sequential images of the inner wall of cooling holes with different depths by axially moving the CCD, using depth from focus methods to get the 3D profile of the inner wall of cooling holes, the detailed measurement system is illustrated in Figure 22, whereas it cannot provide specific dimensions of the inner wall. The 5-axis visual measurement platform organizes the measurement trajectory based on the 3D digital model of blades, achieving a repeatability accuracy for hole diameter within −10 μm to +10 μm and for hole center coordinates within −12 μm to +12 μm. Building on this research, Bi [81] proposed a method for synthesizing axis of cooling hole using a 3D point cloud. By fitting the annular point cloud of inner wall to obtain the center coordinates and then fitting the center coordinates at different depths into a straight line, the axis of cooling hole is finally established. To simplify, convert the calculated direction vector of axis into the angle between the vector and the coordinate axis, as well as the angle between the vector projection and the coordinate axis. The angles show a repeatability error within 0.3°.

Wang Cheng [40] utilized a 5-axis optical CMM to measure cooling holes. They employed a CCD to capture images of cooling holes and calculate the diameter and center coordinates of the holes. To validate the measurement accuracy, the measuring platform was used to measure a simulation specimen (standard disc), and limit error is 0.024 mm for diameter and 0.042 mm for positional accuracy. Nevertheless, since the measurement process requires continuous adjustments relative position of the CCD and cooling hole to align the hole axis, it relied on manual experience and subjective visual judgment, which can potentially affect the precision of the measurement results.

Liao Tao [82] modelled a digital virtual specimen of blades by extracting blade profile parameters, including the number and position information of cooling holes. They developed specialized software based on the digital virtual specimen, enabling real-time prediction and compensation of drilling positions during the manufacturing, according to the extent of blade deformation. The simulation results and experiment results demonstrate that the positioning errors of cooling holes are 1.34 μm and 4.25 μm, respectively, proving this prediction method can satisfy the cooling efficiency requirements. Zhang Min [83] researched the issue of axial inspection of cooling holes. They proposed an inspection scheme for the axial direction based on an improved Gaussian mapping algorithm. Using a line laser scanner, the high-precision point cloud is obtained to extract the axial feature parameters of cooling holes. The experiment results confirm that this method achieved an extraction precision of 0.6290°.

Based on the above studies, Table 2 organizes and summarizes the main measurement technologies for cooling holes, outlining the measurement parameters of each technology along with their advantages and disadvantages.

### 4.3. Difficulties in Application of Digital Measurement Technology for Cooling Hole

Based on the background mentioned above, this section summarizes the technological difficulties in applying digital measurement technologies to cooling holes:

- Microscale and Complex Geometries

Cooling holes typically have small sizes, large depth-to-diameter ratios, and complex axial angles. It is challenging for light to illuminate the cooling holes and reflect to the camera, which restricts ordinary visual observation and visual inspection of orifice areas. These geometric characteristics result in significant inconvenience to the measurement.

- Requirements for High Precision and High Resolution

In addition to vital quality indicators such as the diameter and axis orientation of cooling holes, which evidently impact cooling efficiency, some minute damages like recast layers and microcracks are hard to measure accurately. Traditional measurement equipment and technologies often struggle to achieve the aim of precise measurement, failing to meet the requirements for high precision and resolution.

- Standardization and Uniformity

The absence of uniform quality assessment standards and standardized measurement methods can influence the accuracy and reliability of cooling hole measurement results. Different measurement devices and technologies may generate varying data types, posing an adverse to ensuring data consistency and comparability across different measurement platforms and methodologies.

- Time Efficiency

While ensuring measurement accuracy, the time cost of measurement must also be considered. Given that a single blade has hundreds of cooling holes and an aero-engine requires several hundred blades, manual inspection methods are impractical for large-scale production. Therefore, efficient measurement methods are crucial to meet large-scale production requirements without sacrificing high precision.

### 4.4. Shortcoming of Current Measurement Technology for Cooling Hole

The shortcomings of current measurement technologies are analyzed in this section.

- Single Measurement Quality Indicators

Research on non-contact measurement primarily focused on the geometric shape of the orifice, with less emphasis on positional accuracy and inner wall manufacturing quality. Contact measurement can measure the geometry of the inner walls, but fails to reflect manufacturing quality and micro defects accurately. So far, single-measurement methods can only assess the geometric shape, positional accuracy, or inner wall quality of cooling holes and cannot measure all quality indicators in a single operation.

- Absence of Digital Measurement Strategy

Most current research primarily focuses on individual cooling hole measurement methods and enhancing measurement precision. The absence of comprehensive measurement planning, position and orientation modelling of blades and measuring equipment, digital modelling of cooling hole geometric features, and the construction of cooling hole measurement platforms are notable. It is urgent to fulfil the aim of efficient and precise automated measurement.

- Absence of Error Analysis

In the actual measurement process of cooling holes, due to the wide distribution of holes and significant variation in axial orientation, measuring each cooling hole individually requires the measurement devices to vary the position and orientation continuously. This process may induce cumulative errors, affecting the final measurement results. Currently, there is no specialized research addressing this issue.

- Absence of Comprehensive Multi-Hole Measurement

Discrete hole characteristics cannot estimate the influence of positional accuracy on cooling performance from the perspective of cooling hole distribution. At the moment, there is limited research on combining multiple discrete hole measurement data to obtain a comprehensive cooling hole distribution pattern on the blade.

## 5. Summary and Outlook

As the requirements of aero-engine performance continuously increase, the development of cooling technologies, including the optimization and improvement of shaped cooling holes and manufacturing processes, presents problems for precise measurement. This paper focuses on analyzing various cooling hole measurement methods with different principles, comparing their advantages and appropriate applied scenarios, and identifying the difficulties and current technological drawbacks. This paper is of significant value for future research.

Future work in this field should be developed in the following directions:

- Establishing a Comprehensive Technical Framework for Cooling Hole Measurement

This framework involves generating measurement planning strategies based on digital blade models, implementing automated digital measurement based on intelligent equipment, and conducting data analysis and quality assessment. The ultimate goal is to enhance the level of automation and efficiency in cooling hole measurement.

- Optimizing Vision Measurement Methods

For vision-based measurement technologies, the design of the optical path and the selection of measurement equipment and construction of the measurement system should be refined. These optimizations point to improving the precision of measurement results and operation efficiency. Enhancements should include advanced image processing algorithms, calibration technologies, and the utilization of higher resolution CCD.

- Focusing on Comprehensive Cooling Hole Quality Indicators

Future research should not only concentrate on measuring discrete hole geometrical features, but also pay attention to a comprehensive study of the overall distribution of cooling holes and the manufacturing quality of inner walls. By employing composite measurement methods and integrating results, a 3D digital model of the blade can be established. This model will facilitate analysis of the relationship between cooling performance and cooling hole distribution, as well as their geometrical features and manufacturing quality. Understanding these relationships is beneficial for optimizing blades’ design and manufacturing processes to enhance cooling efficiency.

- Analyzing and Assessing Errors in Cooling Hole Measurement Systems

The sources and quantity of error in cooling hole measurement systems should be analyzed and evaluated in detail. Implementing software algorithms helps mitigate the impact of the errors and enhances measurement precision consequently. This aspect should be harmonized with the efficiency of automated measurement equipment to balance accuracy against efficiency. Application strategies should include developing advanced calibration technologies, improving data processing algorithms, and integrating real-time feedback mechanisms into the measurement process.

## Figures and Tables

**Table 1:** Improvement in cooling performance against shaped cooling holes [23].

Hole Shape	% Improvement of Cooling Performance
Fan shape	10–40
Conical	15
Console	20
Sister hole	15–23
Compound angle	4–10
Trench shape	15–20

**Table 2:** Comparison of main measurement technology for cooling hole.

Main Measurement Technology		Measured Features	Advantage	Disadvantage
Optical measurement	Light field	Geometrical feature of hole inner wall (partial) Orifice shape and diameter	High efficiency	Limited data
	Image recognition	Orifice shape and diameter	High efficiency	Limited data
	luminous flux	Orifice diameter	High efficiency	Limited data
	3D reconstruction	Geometrical feature of hole inner wall (partial) Orifice shape and diameter	High efficiency	Limited data
Industrial CT		Geometrical feature of hole inner wall	Generalized measurement result	High cost, low efficiency
Infrared imaging		Orifice diameter	High efficiency convenient construction of measurement system	Limited data
Probing measurement	Capacitive probe	Geometrical feature of hole inner wall	Suitable for high depth to diameter ratio hole	Low efficiency, low resolution, limited data
	Fiber probe	Geometrical feature of hole inner wall	Suitable for high depth to diameter ratio hole	Low efficiency, low resolution, limited data
	Laser interferometry	Geometrical feature of hole inner wall	Suitable for high depth to diameter ratio hole	Low efficiency, low resolution, limited data
